# Women’s social networks and use of facility delivery services for uncomplicated births in North West Ethiopia: a community-based case-control study

**DOI:** 10.1186/s12884-017-1626-8

**Published:** 2017-12-28

**Authors:** Kerebih Asrese, Margaret E. Adamek

**Affiliations:** 1 Bahir Dar University, Social Work Department, Faculty of Social Sciences, Bahir Dar, Ethiopia; 2 Indiana University, School of Social work, Indianapolis, USA

**Keywords:** Social networks, Facility delivery, North West Ethiopia

## Abstract

**Background:**

High maternal mortality has remained an unmet public health challenge in the developing world. Maternal mortality in Ethiopia is among the highest in the world. Since most maternal deaths occur during labor, delivery, and the immediate postpartum period, facility delivery with skilled birth attendants is recommended to reduce maternal mortality. Nonetheless, the majority of women in Ethiopia give birth at home. Individual attributes and availability and accessibility of services deter service utilization. The role of social networks that may facilitate or constrain service use is not well studied.

**Methods:**

Community-based case-control study was conducted between February and March 2014 in Jabi Tehinan District, North West Ethiopia. Retrospective data were collected from 134 women who had uncomplicated births at health facilities and 140 women who had uncomplicated births at home within a year preceding the survey. Interviews were held with eight women who had uncomplicated births at health facilities and 11 who had uncomplicated births at home. The quantitative data were entered and analyzed using SPSS for Windows versions 16.0 and hierarchical logistic regression model was used for analysis. The qualitative data were transcribed verbatim and data were used to substantiate the quantitative data.

**Results:**

The results indicated that social network variables were significantly associated with the use of health facilities for delivery. Taking social networks into account improved the explanation of facility use for delivery services over women’s individual attributes. Women embedded within homogeneous network members (Adjusted OR 2.53; 95% CI: 1.26–5.06) and embedded within high SBA endorsement networks (Adjusted OR 7.97; 95% CI: 4.07–12.16) were more likely to deliver at health facilities than their counterparts. Women living in urban areas (Adjusted OR 3.32; 95% CI: 1.37–8.05) and had better knowledge of obstetric complications (Adjusted OR 3.01; 95% CI: 1.46–6.18) were more likely to deliver at health facilities.

**Conclusion:**

Social networks facilitate SBA utilization by serving as a reference for the behavior to deliver at health facilities. These findings inform health professionals and other stakeholders regarding the importance of considering women’s social networks in designing intervention to increase the proportion of women who deliver at health facilities.

## Background

Since the 1980s, the high level of maternal mortality has been an agenda of global health and development discussions [1]. In the 2000 millennium summit, improving maternal health was identified as one of the Millennium Development Goals to be attained by 2015. Yet maternal mortality has remained an unmet public health challenge, particularly in developing countries. Worldwide, 358,000 women between the ages 15–49 die every year from pregnancy and childbirth-related causes. Developing regions account for 99% of such deaths. Among the developing countries, Sub-Saharan Africa contributed more than half (57%) of the deaths [2].

As in other Sub-Saharan African countries, maternal mortality in Ethiopia is one of the highest in the world [3]. With 14,000 women dying each year, Ethiopia is among the top five countries with the highest number of maternal deaths worldwide [2]. According to the 2011 Demographic and Health Survey, Ethiopia has an estimated maternal mortality of 676 deaths per 100,000 live births (range of uncertainty for the estimates was 541–810) [4]. This estimate is similar to the 2005 estimated maternal mortality rate of 673 deaths per 100,000 live births (range of uncertainty for maternal mortality rate estimates 548–799) [5]. These estimates indicate that maternal mortality remained unchanged despite the millennium development goals.

Since the majority of maternal deaths occur around the time of labor, delivery, and the immediate post-natal period, maternal health programs tend to prioritize skilled delivery care at birth and the management of complications to save women’s lives [6]. The presence of skilled birth attendants (SBAs) at delivery helps to prevent, detect, and manage the major obstetric complications. Timely obstetric care by SBAs saves women’s lives and reduces maternal mortality [7].

In Ethiopia, the rate of delivering babies with the assistance of SBAs is among the lowest in the world. Only 15% of births were assisted by skilled birth attendants in health facilities. Ninety percent of births occurred at home under unhygienic conditions [4]. Improving maternal health and reducing maternal mortality is one of the priority goals within the government of Ethiopia’s successive Health Sector Development Programs [8]. Thus, research is needed to understand the factors that serve as barriers or that contributes to facility delivery service utilization.

Previous studies that assessed maternal healthcare utilization in the country documented individual attributes (age, parity, educational status, household income, and husbands’ educational status) and external environmental barriers (residence and availability and accessibility of services) to service utilization [9–12]. While these studies make important contributions with respect to identification of relevant variables, they do not indicate the condition of service utilization, i. e., whether women used the service to prevent unforeseen complications (normal biology of pregnancy and uncomplicated childbirth) or for curative purposes after certain complications happened. Importantly, the existing studies do not fully explain the social networks--interpersonal, family, and community interactions--that may facilitate or constrain service use.

Studies in other parts of the world demonstrated that social networks, i.e., interpersonal interactions, can facilitate or hinder healthcare utilization [13, 14]. Interpersonal interactions can provide information on institutional details of the health care system, and can alter the demand for services by affecting the perceived usefulness of the available services [15]. Social networks provide social support (both perceived and actual), social influence (norms and social control), social engagement, person-to-person contacts, and access to resources (money, jobs, and information), thus affecting health service utilization [16]. There is no study in Ethiopia known to the writers that investigated the influence of social network variables on facility delivery service utilization, particularly for women with uncomplicated births.

Thus, the main objective of this study was to investigate the contribution of social network variables, in addition to individual characteristics, on facility delivery service utilization. The study was expected to identify factors that facilitate or constrain facility delivery service utilization and thereby inform the design of interventions to increase the proportion of women who deliver at health facilities with the assistance of SBAs.

## Methods

### Study setting and design

A community-based case-control study was carried out in Jabi Tehinan district, Amhara Region, North West Ethiopia. The cases comprised of women who had uncomplicated births (spontaneous vaginal delivery) for a child at a health facility within a year preceding the survey. The controls included women with uncomplicated births for a child at home during the same period.

Administratively, the disstrict is divided into 5 urban and 39 rural kebeles. There are 37 health posts and 12 health centers providing health services for the population including maternal health services. Seven out of 12 health centers have midwifery. The study was conducted within 5 km around these health centers [17].

Maternal health service utilization is at a low status in the district. At the time of the survey, the proportion of women who attended antenatal care service at least 4 times was 6.6%, SBA utilization was 9%, and post-natal service utilization was 35% [17]. Thus, the sample size for the study was determined using single population proportion formula considering 9% proportion, 95% confidence interval with 5% margin of error, and 10% non response rate. The final sample size was 278 mothers.

A sequential explanatory mixed methods design was employed [18]. The qualitative data complemented the survey data by providing an interpretive aid to understanding the statistical patterns that emerged. Data collection with both methods was linked by addressing the same substantive issue of the influence of social networks on facility delivery. “The more that the items overlap or complement each other; the more that the mixed methods can be part of a single study” [19]. Integration occurred at the data interpretation stage and in the discussion [18, 19]. This design is useful for explaining relationships and/or to elaborate, illustrate, and clarify the quantitative findings [20, 21].

### Study procedure

We initially developed the survey questionnaire in English and then translated into Amharic to ease understanding. Prior to the main study, a pretest was conducted among 25 women (10 women had uncomplicated facility deliveries and 15 women had uncomplicated home deliveries) not selected for the study and the necessary adjustment in language and content was done. The first author and two research assistants supervised the main data collection process. The first author also collected the qualitative data after the survey was completed.

### Participants of the study

Ever-married women in the reproductive age group (15–49) who had uncomplicated births (spontaneous vaginal delivery) within a year preceding the survey and had living children participated in the study. The participants were divided into two groups based on place of delivery. A list of women who delivered at health facility within the preceding year was obtained from the delivery registration books from health institutions providing delivery services. Those women who had uncomplicated deliveries were selected for inclusion in the study and assigned as cases. The control group were women who had uncomplicated deliveries at home. Two kebeles (lowest administrative unit) within the vicinities of the health institutions were identified in the district. Lists of women who delivered within a year in those kebeles were obtained from the health posts’ vaccination registration books in each selected kebele. The names of babies were randomly selected and their mothers were approached and asked to participate in the study.

Two hundred seventy-eight respondents-138 cases (women who delivered at health facilities) and 140 controls (women who delivered at home) participated in the quantitative phase of the study. A sub-set of 19 women, 8 who delivered at health facilities and 11 who delivered at home, also participated in the qualitative phase of the study.

### Variables

The primary dependent variable was place of delivery, a dichotomous variable indicating the place of delivery for participants’ recent born children- at a health facility or at home (coded 0 = home delivery and 1 = facility delivery). The independent variables in the study included a variety of network variables and the respondents’ individual characteristics. The network variables were measured as follows.

- *Network size*: the number of individuals in a woman’s network identified by respondents.
- *Network tie strength:* the strength of the relationship between the respondent and her nominated network members measured in terms of closeness or intimacy using a four point scale (distant = 1, less close = 2, close = 3, and very close = 4). The sum of valid responses was divided by the number of valid responses to compute the summary measure of network tie strength. *Homogeneity of network*: kinship or non-kinship status of the network members. Networks that were comprised of parents and relatives who had biological relationships were considered as kin. The responses were coded as kin = 1, non-kin = 0. The sum of valid responses divided by the number of valid responses was used to compute the summary measure of network homogeneity.
- *Network content*: Network content was the place of delivery endorsement, i. e., respondents’ perception of the advice, opinion, and suggestion of their network members about the place of delivery for their recent born children measured using a three point scale (at home = 1, at a health facility if there would be complication = 2, and at a health facility = 3). The summary measure of SBA endorsement (health facility delivery endorsement) was obtained by dividing the sum of valid responses by the number of valid responses.
- *Geographical dispersion of network:* the neighborhood status of network members measured by responses coded as non-neighbor = 1, and neighbor = 2. The sum of valid responses was divided by the number of valid responses to compute the summary measure of the neighborhood status.
- *Respondents’ individual attributes* **:** socio-demographic and childbearing-related characteristics that might influence delivery service utilization. These variables included age, women’s educational status, husbands’ literacy status, women’s autonomy in household decision-making, knowledge of obstetric complications (measured using seven items with nominal responses), parity, and antenatal care use during their recent pregnancy.

### Operational definitions

#### Uncomplicated birth at health facility

Spontaneous vaginal delivery for a child at a health facility with the assistance of SBAs without using delivery instruments such as vacuum and forceps.

#### Uncomplicated birth at home

Spontaneous vaginal delivery for a child at home and mothers did not visit health facilities for help due to complication or illness during labor, delivery, and post-natal period within a month.

### Data analysis

All returned questionnaires were checked for completeness and consistency of responses manually. After cleaning, the items were coded and entered, in to SPSS for Windows versions 16 for analyses.

One objective of the study was to investigate whether women’s social networks were more important than individual attributes in predicting the use of SBAs during uncomplicated delivery for their recent-born children. Thus, hierarchical binary logistic regression analysis using the Enter method was used [22]. Social network analysis considers the social structural or relational processes as primary and individuals’ attributes as secondary in influencing the observed behavior of individuals [23, 24]. Thus, women’s individual attributes that showed significant associations with the dependent variable in previous studies were entered first in the model and the network variables were entered later. The significance of improvement in predicting the dependent variable was assessed based on the -2log likelihood ratio tests for the first block of the individual attributes and the second block of the network variables [25].

The second objective of the study was to assess the relative importance of the various network variables in predicting facility delivery service utilization. Thus, the *p*-values for the network variables were compared. The variable with the smallest p-value was considered the most important variable in predicting the response variable [26].

Qualitative data were transcribed verbatim. The transcription of data was undertaken on the same date of each interview. The transcript of each interview was read and re-read thoroughly to understand the content and concepts shared in the interview and to look for differences and similarities in the data [27]. Data were then coded and categorized into themes based on the study objectives. This approach is appropriate when the qualitative data were collected to provide further understanding of the quantitative findings [28].

### Ethical consideration

The study received ethical approval from Ethical Review Committee of Bahir Dar University. A month before data collection, we communicated the selected health institutions and ‘kebele’ administrations with formal letters obtained from Bahir Dar University. Verbal consent of individual participants was obtained after being fully informed of the study purpose and procedures. We ensured confidentiality by removing all personal identities from the questionnaire.

## Results

The study included 274 women who had uncomplicated births within a year prior to the survey. One hundred forty (51%) respondents delivered at home and 134 (49%) delivered at a health facility. The respondents ranged in age from 18 to 40 years with a mean age of 27 years. Seventy-five percent of the respondents live in rural areas and about 25% live in urban areas. Among the respondents, 63% were illiterate, 26% completed primary education (grades 1 to 8), and 11% had high school and above education level. About 57% of the respondents had literate husbands (Table 1).

**Table 1:** Percentage distribution of respondents by background characteristics and place of delivery (*N* = 274)

Background characteristics	Home delivery *n* (%)	Facility delivery *n* (%)	Total *N*(%)
Age			
18–23	28 (44.4)	35 (55.6)	63 (22.3)
24–29	67 (53.2)	59 (46.8)	126 (46)
> =30 years	45 (52.9)	40 (47.1)	85 (31)
Residence			
Rural	119 (57.8)	87 (42.2)	206 (75.2)
Urban	21 (30.9)	47 (69.1)	68 (24.8)
Educational status			
No education	96 (55.8)	76 (44.2)	172 (62.8)
Primary (1–8)	42 (58.3)	30 (41.7)	72 (26.3)
Secondary & above (> = 9)	2 (6.7)	28 (93.3)	30 (10.9)
Husband’s Literacy status			
Illiterate	78 (65.2)	44 (34.8)	122 (43.1)
Literate	62 (40.8)	90 (59.2)	152 (56.9)
Parity			
1–2 children	64 (48.9)	67 (51.7)	131 (47.8)
3–4 children	60 (56.6)	46 (43.4)	106 (38.7)
> =5 children	16 (43.2)	21 (56.8)	37 (13.5)
ANC use			
No	43 (70.5)	18 (29.5)	61 (22.3)
Yes	97 (45.5)	116 (54.5)	213 (77.7)
Knowledge of obstetric complications			
Limited knowledge	51 (62.2)	31 (37.8)	82 (29.6)
Better knowledge	89 (46.4)	103 (53.6)	192 (70.1)
Autonomy with household decisions			
Limited participation	10 (58.5)	7 (41.2)	17 (6.2)
Had participation	130 (50.6)	127 (49.4)	257 (93.8)
Total	140 (51.1)	134 (48.9)	274(100)

Close to half (47.8%) of the respondents had 1 or 2 children, 38.7% had 3 to 4 children, and 13.5% had 5 or more children. Nearly 78% of the respondents had ANC check-ups during their recent pregnancy. Seventy percent of the respondents rated themselves as having better knowledge of obstetric complications.

Respondents were also asked about their participation in household decisions such as health care, large purchases, small purchases, and visits to relatives. More than 93% reported that they had participation in deciding on the concerns assessed in the study. Only 6.2% reported that they had limited autonomy to decide on those household concerns.

A greater proportion of women living in urban areas delivered at health facilities than their rural counterparts (69.1% vs. 42.2%). Compared with women who delivered at home, those who delivered at a health facility were more likely to have literate husbands (59.2% vs. 40.8%), were more likely to have ANC follow-ups during their recent pregnancy (54.5% vs. 45.5%), and had somewhat better knowledge about obstetric complications (53.6% vs 46.4%).

Table 2 presents the percent distribution of respondents by network characteristics and place of delivery. The respondents’ network size ranged from 2 to 16 with a mean network size of 9.7 members. The network size of respondents was summarized into three categories based on mean ± 1 standard deviation: 2 to 6 network members, 7 to 12 network members, and 13 to 16 network members. The data revealed that the proportion of women who delivered in health facilities increased with increasing network size (19.7% in the category 2 to 6 network members to 62.9% in the category 13 to 16 network members). Conversely, the proportion of women who delivered at home decreased with increasing network size (80.3% in the category 2 to 6 network members to 37.1% in the category 12 to 16 network members).

**Table 2:** Percentage distribution of respondents by network characteristics and place of delivery for recent-born children (*N* = 274)

Network characteristics	Home delivery *n* (%)	Facility delivery *n* (%)	Total *N* (%)
Network size			
2–6 network members	49 (80.3)	12 (19.7)	61 (22.3)
7–12 network members	65 (45.5)	78 (54.5)	143 (52.2)
13–16 network members	26 (37.1)	44 (62.9)	70 (25.5)
Network tie strength			
Weaker tie	59 (47.2)	66 (52.8)	125 (45.6)
Strong tie	81 (54.4)	68 (45.6)	149 (54.4)
Homogeneity of network			
Less homogeneous	63 (55.8)	50 (44.2)	113 (41.2)
Homogeneous	77 (47.8)	84 (52.2)	161 (58.8)
SBA endorsement			
Low	95 (71.4%)	38 (28.6)	133 (48.5)
High	45 (31.9%)	96 (68.1)	141 (51.5)
Network neighborhood			
Non-neighbor	55 (50.5)	54 (49.5)	109 (39.8)
Neighbor	85 (51.5)	80 (48.5)	165 (60.2)
Total	140 (51.1)	134 (48.9)	274 (100)

About 54% of respondents reported strong tie networks and 45.6% reported weaker tie networks. Among respondents embedded in weaker tie networks, 47.2% delivered at home and 52.8% delivered at a health facility. Of those respondents embedded in strong tie networks, 54.4% delivered at home and 45.6% delivered at a health facility. Women who delivered at home had stronger tie network scores than women who delivered at health facilities (54.4% vs. 45.6%). Nearly 60% of the respondents had a homogeneous network and 41% had a less homogeneous network. Among women embedded in homogeneous networks, 47.8% delivered at home and 52.2% delivered at a health facility.

Furthermore, 48.5% of women were embedded within low SBA endorsement networks and 51.5% were embedded within high SBA endorsement networks. Of those women who were embedded within low SBA endorsement networks, 71.4% delivered at home and 28.6% delivered at a health facility. Among respondents with high SBA endorsement network, 31.9% delivered at home and 68.1% delivered at a health facility. Thus, women who delivered at a health facility had larger SBA endorsement scores than those who delivered at home.

### The influence of social networks on facility delivery

The main intent of the research was to understand whether the women’s social networks or their individual attributes were more important in predicting the use of SBAs during uncomplicated delivery of their recent-born children. The -2log likelihood in model I with the individual attributes added was (313.836). The -2log likelihood in model II after the network variables were added was (245.685), indicating a reduction in error associated with the inclusion of the network variables in predicting facility delivery service utilization. The chi-square analysis in model II showed a significant difference between the -2log likelihood ratio at model I and -2log likelihood ratio at model II (χ2 = 134.116, df = 15, *N* = 274, *p* < 0.001). The overall correct classification of respondents into home delivery and facility delivery improved from 69.3% model I (that included women’s individual attributes) to 77.4% model II (that included the individual attributes and the network variables).

The results indicate that there was a statistically significant improvement in predicting facility delivery use with the network variables i.e., the network variables were important predictors for distinguishing between respondents who delivered at home and those who delivered at a health facility after controlling for women’s individual attributes. Women’s social network variables better predicted facility delivery service utilization for their recent-born children than their individual attributes.

The odds of facility delivery was 1.29 (95% CI: 1.16–1.45) times higher for every one member increase in network size. The odds of women who were embedded within a homogeneous network members to deliver in a health facility was estimated to be 2.53 (95% CI: 1.26–5.06) times higher than women embedded within less homogeneous network members. Women who were embedded within high SBA endorsement networks were 7.97 (95% CI: 4.07–12.16) times more likely than women who were embedded within low SBA to deliver in a health facility.

As indicated in Table 3, women’s individual attributes (educational level, residence, and knowledge of obstetric complications) significantly predicted facility delivery. The odds of women who had high school and above education level to deliver at a health facility was 8.07 (95% CI: 1.36–17.64) times higher than for illiterate women. The odds of women who had good knowledge of obstetric complications to deliver in a health facility was 3.01 (95% CI: 1.46–6.18) times higher than women who had limited knowledge about the complications. The odds of urban women to deliver at a health facility was 3.32(95^CI: 1.37–8.05) times higher than their rural counterparts.

**Table 3:** Hierarchical Logistic Regression analysis of women’s individual attributes and their social network variables predicting facility delivery (*N* = 274)

Variables	Odds ratio (95% confidence interval)	
	Model I	Model II
Age of respondents	.92(.82–1.14)	.94(.86–1.03)
Educational level		
No education®	1	1
Primary (1–8)	.95(.29–3.12)	.54(.25–1.19)
High school and above	3.79 (2.31–23.04)*	8.07(1.36–17.64)*
Husband’s educational status		
Illiterate®	1	1
Literate	2.20(.62–5.05)	1.70((.84–3.43)
Residence		
Rural®	1	1
Urban	2.75(.86–8.77)	3.32(1.37–8.05)**
Parity		
1–2 children®	1	1
3–4 children	.22(.04–1.09)	.85(.38–1.92)
> = 5 children	1.91(.08–4.36)	2.35(.57–9.73)
Use of ANC		
No®	1	1
Yes	4.33(.85–2.13)	1.86(.83–4.17)
Knowledge of obstetric complications		
Limited ®	1	1
Better knowledge	2.31(.70–7.63)	3.01(1.46–6.18)**
Household autonomy	.63(.21–1.92)	1.47(.61–3.52)
Network size	–	1.29(1.16–1.45)***
Network tie strength		
Weak®		1
Strong	–	.73(.35–1.55)
Network homogeneity		
Less homogeneous ®		1
Homogeneous	–	2.53(1.26–5.06)**
SBA endorsement		
Low ®		1
High	–	7.97(4.07–12.16)***
Network neighborhood		
Non-neighbor®		1
Neighbor	–	1.65(.85–3.36)
Model G^2^ (−2log likelihood)	313.836	245.685
Degree of freedom	10	15
Changed Chi-square	95.731***	134.116***

® reference category, *P < .05, ** *P* < .01, *** *P* < .001

The results also revealed the relative importance of network variables in predicting facility delivery service utilization. Network size and SBA endorsement had the smallest p –values (*p* < 0.001) followed by the homogeneity of network members (*p* < .01).Thus, network size and SBA endorsement by network members were more important in predicting health facility delivery service utilization than other network variables.

Informants in the qualitative study described that social networks have great influence during pregnancy, labor, and delivery. Their social networks often give them emotional support and assisted them in household chores such as fetching water, grinding cereals, preparing *‘tella’*, and shopping during pregnancy. During labor and delivery, members of their social networks were praying-*mariam, mariam*, to shorten the duration of labor and to make the delivery smooth.

Some informants shared that their social networks facilitated transport to the health facility for delivery. For example, Tiringo shared: “we are living away from both relatives. When labor started, I told my husband. He immediately called on a transport and we went to the health facility.” Tatu similarly reported:

It was my first child. I was new for labor pain. When I felt unusual pain, I told my husband. He told my mother what I felt. She came …. She guessed that it was labor. Of course, it was…. After a while, other relatives heard the event. There was praying. The labor was long. … My mother wanted me to deliver at home. However, my husband called an ambulance and we went to a health facility for delivery (Tatu, parity 1).

Some informants shared that their place of delivery for their recent-born children was suggested by their network members. Tatu delivered her child at a health facility though her mother preferred that she deliver at home:

It was my first birth. I stayed long on labor, about 12 h. People were praying all the day expecting that I would deliver soon. Around sunset, my husband proposed that I should go to a health facility. He expected there might be a complication. Mother asked, ‘why?’ She said, ‘I delivered all my children at home…. There is no need to go to health facility.’ Other relatives supported his idea…. Thus, I delivered at a health facility.

The informant’s narrative demonstrates that she was not the primary decision-maker about where to deliver her child. Some informants shared that they planned to deliver at a health facility because they had information about the importance of facility delivery from different sources. For example, Zinash stated:

I knew the importance of facility delivery even before pregnancy. Sometimes, when someone delivered recently, neighbors and relatives come together and chat about her delivery. In those occasions, we used to chat about what she said, what her husband said, and on the overall birthing process. I think I learned a lot from such discussions. During recent pregnancy, just a month or so before, my friend delivered at a health facility. I accompanied her to the health facility. I observed …. Even, she joked at me that the next will be my turn. When labor started, I went to health facility. My husband and a few other people accompanied me (Zinash, parity 1).

Zenebu and Shita both shared that they delivered at a health facility because they worried about the absence of network members who would assist them at home during delivery. Zenebu shared that:

We are living in new residence away from both relatives. Around the date of delivery, my husband was not at home. I was living with my sister. We did not have relatives in the new residence. I worried a lot. I was thinking who will assist me during the pain. I knew health facilities provide delivery services. During ANC follow-up, the HEWs advised me to deliver at a health facility. Thus, when labor started, I went to the health center for delivery (Zenebu, parity 2).

The informant’s narrative indicated that she believes that social networks (relatives and neighbors) are important to support/assist women during labor and delivery. Because of the absence of relatives and neighbors who could assist her during labor and delivery, she went to the health facility.

Some informants delivered at home because the majority of their network members preferred them to deliver at home. As Aster shared:

When labor started early in the morning, relatives and neighbors who heard the event came to our home. Everybody was praying-*mariam*, *mariam*, *mariam.* My mother became more concerned about the event. … she suggested that I should go to health facility for delivery. However, other people did not support her. They argued, it was not long time since labor was started. There was no sign of any difficulty. The doctors will do nothing in the normal condition; she will lie on the back and they will say push. It is St. Mary who will bring positive outcome. Let us pray to St. Mary together. In the afternoon, I delivered at home (Aster, parity 4).

## Discussion

This study investigated the influence of women’s social networks and their individual attributes on place of delivery during uncomplicated births in Amhara Region, North West Ethiopia. The study also assessed the relative importance of social network variables on place of delivery. The study employed a cross-sectional case control mixed methods design. Data were retrospectively collected from two groups of women: those who had uncomplicated deliveries at health facilities and those who had uncomplicated deliveries at home.

There are some limitations to the study. Since women were asked to recall retrospectively their network members and their roles, there may be memory lapses that affect the accuracy of recall. Women may report inaccurate data or they may give responses which they believed to be expected or acceptable. Although limitations are inherent, the findings are useful to communities of similar settings. Social networks may influence SBAs utilization during uncomplicated birth in diverse geographical areas where the behavior is not well practiced.

The results revealed that social network variables predict use of health facilities for delivery of uncomplicated births more than individual attributes alone. The hierarchical logistic regression indicated that there is a hierarchical relationship between the social network variables and place of delivery. The network variables significantly reduced the error in predicting the use of facility delivery services. The use of facility delivery services was better predicted with the addition of network variables than women’s individual attributes alone.

Though literature on the influence of social networks on the use of facility delivery services is limited, the significance of social networks for health service utilization has been extensively documented by earlier studies [14, 15, 29–31]. Social networks play important roles for health service utilization by recognizing the need for services, providing information about available services, and mobilizing support to use the services [15]. Social networks are important sources of information for individuals to learn about and evaluate the behavioral strategies to maintain their health [32].

A majority of the informants in the qualitative study shared that their social networks played important roles during pregnancy, labor, and delivery. During pregnancy, the network members provide emotional support, gave advice about health, and assisted with household chores. During labor and delivery, the network members participated in praying and assisting during the birthing process. Some of the informants who delivered at a health facility indicated that their network members told them about facility delivery and the quality of delivery services at the health facility. Others described that their social networks helped them to reach the health facility for delivery.

Among the network structural variables considered in the study, network size and network homogeneity were significantly associated with SBA utilization. The odds of pregnant women delivering at a facility increased with increasing network size. Similarly, the results revealed that women embedded in homogeneous networks (many of the network members are kin) are more likely to deliver at a health facility than women embedded in heterogeneous networks. The results are consonant with earlier studies that reported significant associations between social network structure variables and maternal health services utilization [33, 34]. In their study of the roles of social network structural variables on prenatal care utilization to prevent unforeseen health complications, Clair et al. reported that women embedded within large networks where many of the network members were relatives (homogeneous) were more likely to use the service [32]. Social network structure and content were also found important in explaining women’s service utilization in rural Kenya [34].

Social network content (perceptions of women about the opinion and advice of their network members about place of delivery for their children) was significantly associated with utilization of facility delivery services. Women who perceived that their network members suggested facility delivery were more likely to deliver at a health facility. This result corroborates earlier research reports [34, 35] that social network content was significantly associated with facility delivery.

Though the overall hierarchical regression model result indicated that social network variables predicted the odds of facility delivery better than women’s individual attributes alone, the Wald test indicated significant associations between some of women’s individual attributes and utilization of facility delivery services. The results depicted that women’s educational status, residence, and knowledge of obstetric complications significantly predicted use of facility delivery services. Compared to rural women, urban women were more likely to deliver at a health facility (42.2% vs. 69.1%). Women with relative better knowledge of obstetric complications during labor and delivery were more likely to deliver at a health facility than women with limited knowledge (53.6% vs. 37.8%). Women with high school and above educational status were also more likely to deliver at a health facility than women with no education (93.3% vs. 44.2%). The results are consistent with several studies [36–38], including studies specific to maternal health care utilizations in Ethiopia [4, 5, 11, 12].

Previous studies also reported the significant roles of ANC for facility delivery service utilization. Children are more likely to be delivered at health facility from mothers who had ANC visits than from mothers without ANC visits [4, 5, 9]. Nevertheless, in this study, when the effects of other predictor variables were controlled in the multivariate analysis, ANC visits failed to significantly predict facility delivery (*p* > 0.05). A possible reason for this result may be the content of the ANC counseling provided at health facilities. Women may not get appropriate counseling during the ANC sessions about the benefits of delivering at health facilities. For example, one of the informants described that around the end of the gestation period, she felt discomfort and she went to a health facility for a check-up. The professional told her that the pregnancy was in good condition; and she subsequently she delivered at home. She explained that, “Since I was told that all are at good condition, why should I go to health facility for delivery?”

## Conclusion

In Jabi Tehinan district, more women who delivered at home than those delivered at health facility were embedded within strong tie networks (54.4% vs. 45.6%). Nearly 48% of women who delivered at home and about 52% of women delivered at health facility had network members comprised of mainly homogeneous (kinship) members. Greater proportion of women who delivered at health facility than those who delivered at home perceived that their network members approved of their facility delivery for their recent born children (68% vs. 32%). Neighborhood status of network members for both groups of women was found comparable.

The results revealed that social network variables predicted facility delivery service utilization during uncomplicated delivery better than respondents’ individual attributes alone. Network size was positively associated with facility delivery service utilization. Women embedded within homogeneous networks were more likely to deliver at a health facility. Women who perceived that their network members endorsed facility delivery were more likely to deliver at a health facility. Informants described that their social networks provided them with information about facility delivery services and the quality of facility delivery services and assisted them in reaching the health facilities for delivery.

Women’s individual attributes- residence, educational status, and knowledge of obstetric complications--were significantly associated with facility delivery. Urban women, women with high school and above educational status, and women with relatively better knowledge about obstetric complications were more likely to deliver at a health facility.

Overall, the results indicated that social networks may facilitate or constrain facility delivery service utilization. Stakeholders working to improve uptake of facility delivery service utilization can work with social networks as a means of entry for interventions.The concerned bodies may work with different traditional social organizations such as *mahiber, iquib, idir*, and church meetings where people come together for different social objectives.

## Abbreviation

SBA: Skilled birth attendants
